# Assessment of the Accuracy of Two Different Dynamic Navigation System Registration Methods for Dental Implant Placement in the Posterior Area: An In Vitro Study

**DOI:** 10.3390/jpm13010139

**Published:** 2023-01-10

**Authors:** Tai Wei, Feifei Ma, Feng Sun, Yu Ma

**Affiliations:** First Clinical Division, Peking University School and Hospital of Stomatology, Beijing 100034, China

**Keywords:** dynamic navigation system, U-tube registration, cusp registration, dental implant placement

## Abstract

Purpose: To compare the U-tube and cusp dynamic navigation system registration methods in the use of dental implant placement, and to assess the influence of the location of missing teeth on these registrations. Methods: 32 resin mandible models and 64 implants were utilized, with implants being placed using one of the two registration methods selected at random. Accuracy was measured through the superimposition of the final and planned implant positions. Angular deviation, 3D entry deviation, and 3D apex deviation were analyzed. Results: The overall mean 3D deviation was 1.089 ± 0.515 mm at the entry point and 1.174 ± 0.531 mm at the apex point, and mean angular deviation was 1.970 ± 1.042 degrees. No significant difference (*p* > 0.05) was observed when comparing these two registration methods. However, the U-tube method showed significant difference when assessing the location of missing teeth (without distal-extension absence and distal-extension absence), whereas cusp registration was unaffected. Conclusions: Both the U-tube and cusp dynamic navigation system registration methods are accurate when implemented in vitro. Besides, the cusp registration technique can also overcome several of the limitations of the U-tube approach and the accuracy of it was not influenced by the location of the missing teeth, highlighting it as a method worthy of further clinical research.

## 1. Introduction

Apart from the biological and microbiological factors, the accuracy of the implant position is essential when performing dental implant restoration, as improper implant site selection can increase the short- and long-term risks of postoperative complications [1,2,3,4]. As dynamic navigation system (DNS) approaches continue to be developed and refined, the accuracy of these methods has remained an area of active study. Several reports published to date have confirmed the accuracy of these dynamic computer-assisted surgery systems, which have been reported to decrease the incidence of sinus perforation or inferior alveolar nerve injury during drilling [5,6,7]. Each operative deviation has the potential to affect the accuracy of dental implant placement in the context of DNS-guided dental implant surgery; therefore, accurate registration between the actual and virtual implant areas is essential [8]. The goal of registration is to define the spatial relationship between the operation area coordinate system and the virtual coordinate system, with any given point in the operative area corresponding to a matched X-Y-Z coordinate in the computerized 3D image [9]. Following proper registration, the tip of the operative instrument can be monitored in real-time, allowing for the accurate visualization of its path, position, and the surrounding oral anatomical structures. Operators can thereby adjust instrument position to ensure operative accuracy.

Registration methods developed to date include the use of bone markers such as bone-implanted screws [10], occlusal splints [11], face frame [12], laser surface [13,14,15,16], and anatomical marker registration [17]. The occlusal splint method is the frequently utilized registration approach with accurate, simple, and minimally invasive properties [18,19,20,21,22]. Different DNSs employ different types of occlusal splint, such as U-tube splints, which leverage a tooth-supported reference plate. However, there are some limitations to occlusal splint registration, including long-term storage deformation, accurate positioning difficulties, the need to occupy some amount of dentition space, and the potential to impact fixed device positioning. Cusp registration is a form of anatomical mark registration utilizing the cusp or fossa of the teeth in the same jaw with the missing teeth as a registration marker point. This registration technique does not require the use of additional physical devices and is not disturbed by the soft tissues, thus overcoming some of the limitations of U-tube registration.

The present study was designed to compare the accuracy of cusp registration and U-tube registration in the use of dental implant placement, in order to establish a better registration approach suitable for clinical use.

## 2. Materials and Methods

### 2.1. Study Design

**Figure 1:**
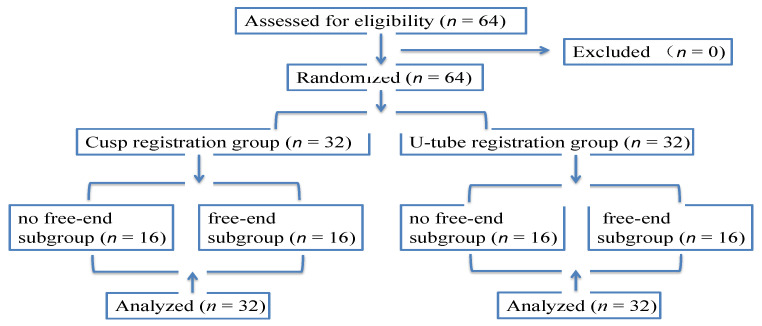
Consort flow diagram showing the grouping of this study.

The in vitro study was carried out between February 2022 and April 2022 at First Clinical Division, Peking University School and Hospital of Stomatology. The U-tube and cusp registration techniques were compared with implants being placed by use of the Dcarer DNS system (Version 2; DHC-D12, Suzhou Digital-health care Co., Ltd.^®^, Suzhou, China). A defined workflow was followed when constructing and executing this study [23,24] (Figure 1). A total of 32 models (64 implants) were selected, including 16 (32 implants) in the test group and 16 (32 implants) in the control group. Each group was subdivided into two subgroups (no free-end and free-end subgroup) and each subgroup had 16 implants. 

The sample size was calculated by using the Clin Epi software (Version 1; Peking University Third Hospital, Beijing, China). Considering a power of 0.90, an alpha value of 0.05, and the results obtained by a prior report [19], a sample size of 64 implants (32 each group) was calculated to be necessary for this analysis.

### 2.2. Model Preparation

**Figure 2:**
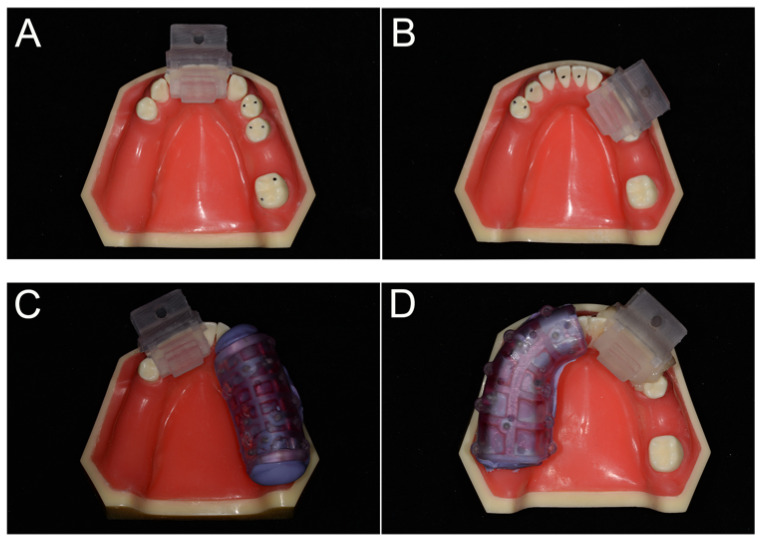
The model of the experiment and the grouping. (**A**) the cusp registration group, without distal-extension absence subgroup; (**B**) the cusp registration group, distal-extension absence subgroup; (**C**) the U-tube registration group, without distal-extension absence subgroup; (**D**) the U-tube registration group, distal-extension absence subgroup.

Sixty-four dental implants (Straumann φ 4.1 mm, BLT, RC,10 mm; Institut Straumann AG, Basel Switzerland) were placed in thirty-two partially edentulous mandible models (Dongguan Liyue Model Science Inc., Dongguan, China) that were constructed specifically for this study using a single template (Figure 2A). The right side of this model missed the first molar (without distal-extension absence), and the left side with three teeth (second premolar, first molar, and second molar) missed (distal-extension absence). The hardness of the model was similar to that of type II bone. An oral surgeon with more than 10 years of clinical experience in implant dentistry and more than three years of experience in using DNS performed all implant placements.

**Figure 3:**
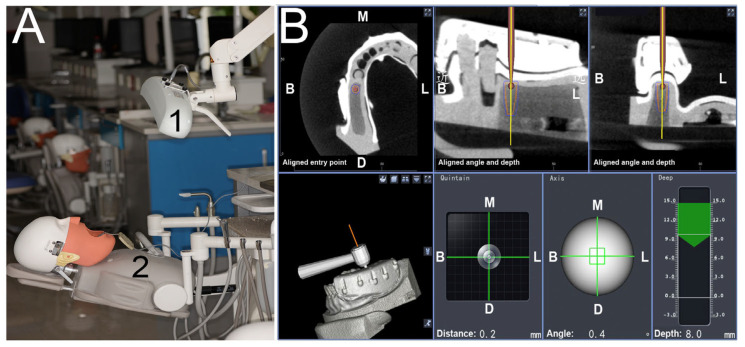
The dynamic navigation system. (**A**) Dcarer^®^ work diagram: 1-infrared receiver (optical camera), 2-reference board; (**B**) Dcarer^®^ software interface during surgical procedures. B: Buccal, L: Lingual, M: Mesial, D: Distal.

The cusp and U-tube registration approaches were, respectively, assigned to the test and control groups. The models were placed in a preclinical learning dental simulator (A-dec Simulator, A-dec Inc., Newburg, MO, USA) with limited mouth opening and a latex face to limit visibility and to mimic facial soft tissues in an effort to better simulate the real clinical situation (Figure 3A).

### 2.3. Treatment Preparation and Surgical Procedures

Prior to implant placement, cone-beam computed tomography (CBCT) scans were taken for all models (Carestream 9300, Carestream Health, France; 75 kV; 4 mA; field of vision: 10 × 10 cm; slice thickness: 180 um). For the U-tube registration group, a U-tube with radiolabeled spots was fixed in the area of the missing teeth using silicone rubber (DMG Dental Products, Hamburg, Germany) during CBCT imaging (Figure 2C,D). No additional device was inserted during CBCT imaging for samples in the cusp registration group. The CBCT DICOM data were uploaded to the navigation system software (Dcarer^®^) and its planning utilities were used to define the dental arch and the position of each implant. A single individual familiar with the software performed all preoperative planning.

**Figure 4:**
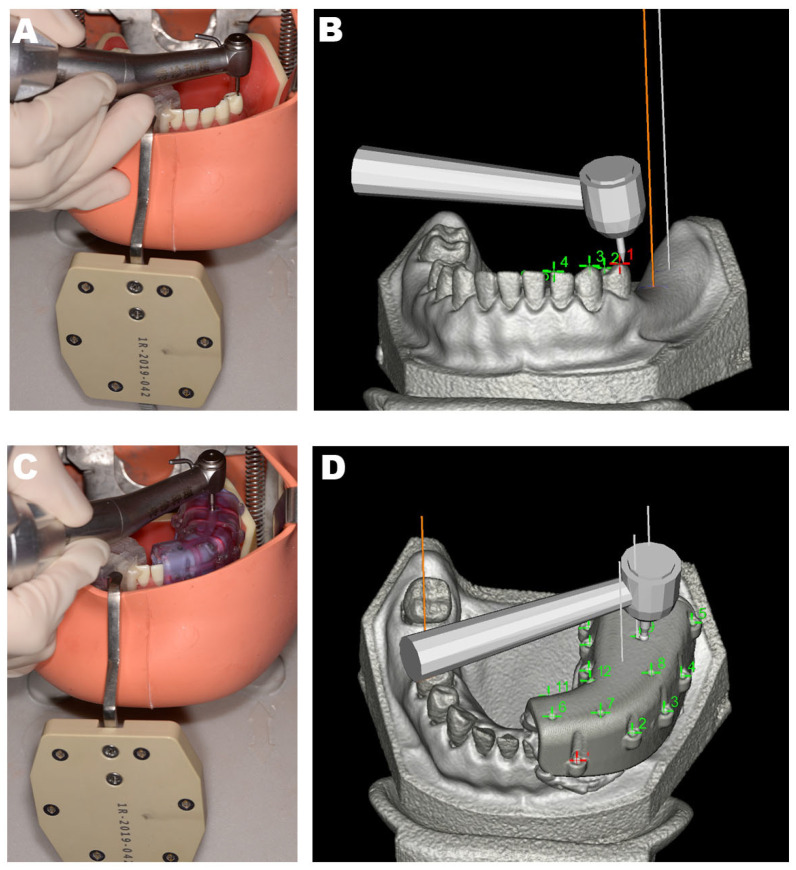
The process of registration. (**A**) cusp registration in oral; (**B**) cusp registration in software; (**C**) U-tube registration in oral; (**D**) U-tube registration in software. The red and green points in Figure (**B**,**D**) are all the points that can be registered. Red one is the point that the mouse clicks in the software.

The implant handpiece and the connecting line of the reference board were matched with the Dcarer^®^ system navigation device, and the infrared receiver (optical camera) of the dynamic navigator was placed above the model (Figure 3A). The reference board was then calibrated in accordance with software guidance, and the infrared connection was adjusted. The reference board was then fixed on the same dental arch with a proper splint and self-curing resin. The implant handpiece with the optical device, the reference board, and the infrared receiver were arranged to remain in a straight, unobstructed path. Cusp or U-tube registration was then conducted (Figure 2). For U-tube registration, the U-tube that was worn during the preoperative CBCT imaging was again worn, and the navigation implant handpiece was used to click the pit on the U-tube with the drill. Six pits were selected for registration in this study. For cusp registration, the cusp or fossa of teeth in the same jaw with the missing teeth were selected in place of the pits selected in the U-tube approach (the points selected as shown in Figure 2A,B. By calculating the distance of the given position from the reference point, point-to-point registration was then conducted to appropriately identify and locate the operative area (Figure 4).

A type 15# scalpel blade was used to generate a crestal incision, after which an elevator was employed to form a soft tissue flap. Drill axis and tip calibration were conducted prior to the initiation of drilling, and implant placement was performed using the recommended drilling protocol. From the start point fixing to the completion of implant placement, the full dynamic navigation operation system was utilized (Figure 3)

### 2.4. Outcome Measures

**Figure 5:**
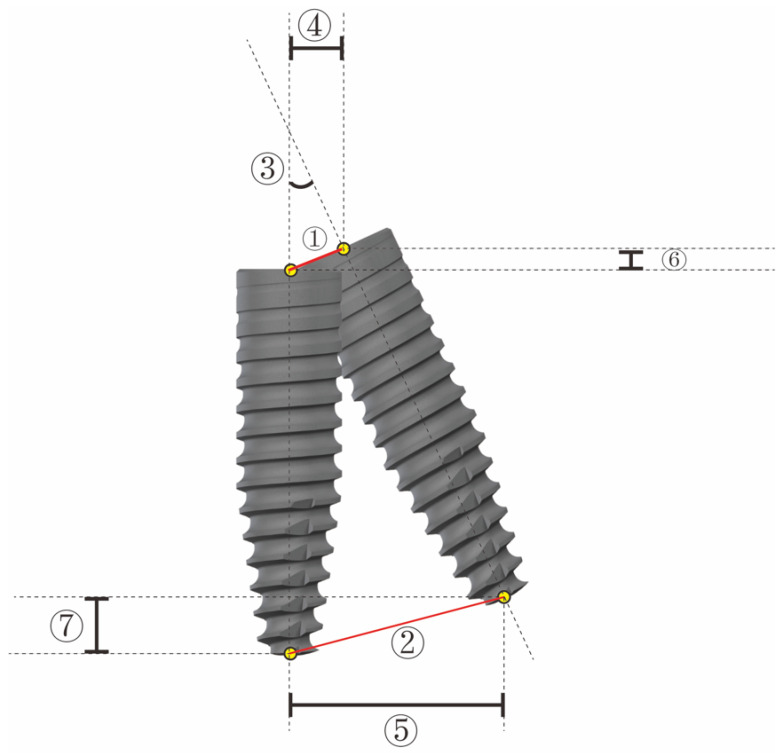
Parameters used to analyze deviations between the planed and inserted implants. ① entry deviation (3D deviation in the coronal aspect of the alveolar ridge); ② apex deviation (3D deviation in the apical area of the implant); ③ angular deviation. ④ entry Horizontal deviation ⑤ apex Horizontal deviation ⑥ entry Depth deviation ⑦ apex Depth deviation.

The accuracy of these two registration techniques was assessed by conducting a second CBCT scan of each model following implant placement using an identical setup to that used for the first scan. The preoperative and postoperative CBCT images were then overlaid by an independent researcher using the Dcarer^®^ dynamic navigation accuracy verification software (Version 2; DHC-D12, Suzhou Digital-health Care Co., Ltd.^®^, Suzhou, China), enabling the comparison of the planned and actual final position of the dental implant. For each implant, the following variables were compared between the preoperative virtual plan and the actual final implant position: angular deviation, entry deviation (3D deviation in the alveolar ridge coronal aspect), and apex deviation (3D deviation in the implant apical area) (Figure 5).

### 2.5. Statistical Analysis

Clin Epi (Peking University Third Hospital, Beijing, China) was used to compute the necessary sample size for this study based upon the selection of angular deviation as the primary outcome variable for these analyses [19,22,24,25]. Mean angulation deviation data were extracted from a prior report [19]. At an alpha value of 0.05 and a statistical power of 90%, a sample size of 64 implants (32 each group) was calculated to be necessary for this analysis. 

All data were recorded and analyzed by a researcher blinded to sample grouping using SPSS (SPSS 23.0, IBM Corp, Armonk, NY, USA). Data were reported as means ± SD, and distribution normality was assessed using the Shapiro–Wilk test. ANOVAs or Student’s *t*-tests were used to compare normally distributed data, whereas non-normally distributed data were compared via the Mann–Whitney U-test. An α = 0.05 significance level was utilized for all analyses. The effect of different registration approaches in area with or without distal-extension and the interaction between these variables were assessed via a two-way ANOVA. If no correlation between the variables was identified, then the corresponding groups were compared for statistically significant variables.

## 3. Results

In total, 64 implants (32 right molars, 32 left molars) were analyzed for this study, with descriptive and bivariate results corresponding to the primary outcome variables for the two registration methods shown in Table 1. The angular deviation, entry deviation, and apex deviation were 2.118 ± 0.940°, 1.172 ± 0.469 mm, and 1.23 ± 0.520 mm in the cusp group and 1.823 ± 0.999°, 1.006 ± 0.567 mm, and 1.119 ± 0.561 mm in the U-tube group, respectively, with no significant difference between groups. When the role of the edentulous area was analyzed in the cusp group (*n* = 16 each), there were no significant differences in these three deviation values when comparing the groups with and without distal-extension absence. In contrast, in the U-tube group, there were significant differences in entry deviation (*p* = 0.026) and apex deviation (*p* = 0.012) between these subgroups (Table 2). 

## 4. Discussion

In this study, the overall mean 3D deviation was 1.089 ± 0.515 mm at the entry point and 1.174 ± 0.531 mm at the apex point, and mean angular deviation was 1.970 ± 1.042 degrees. This result is consistent with the current research related with DNS. One recent meta-analysis research reported that, in the dynamic navigation system group, the pooled weighted mean angular deviation was 3.807, while the pooled weighted mean 3D deviation was 1.305 mm at the apical point and 1.090 mm at the entry point [26]. The placement of implants using a DNS approach has been proved to be more accurate than conventional methods and similar to template-based static guidance [13,27,28,29,30,31]. However, the accuracy also varies depending on the DNS approaches employed [19]. 

A variety of factors can influence error rates in the context of DNS, including operator-, positioning-, registration-, equipment-, navigation software-, and CBCT-related errors. An average error of ~0.5 mm has previously been measured in the process of CBCT data acquisition and processing, directly affecting the accuracy of DNS-based 3D reconstruction [32]. When patients wear a positioning template for preoperative CBCT, this template may warp or cause displacement, and further error can be introduced as a consequence of posture or imaging angle [33]. Some studies have compared the relative accuracy of different DNS equipment systems or examined different countries, analyzing deviations between actual and planned entry points, apex points, and angle values to establish the relative accuracy of these different tools and approaches [18,34,35,36]. The duration and accuracy of the DNS-based implant operation have also been shown to be closely related to the associated surgical learning curve [18,19,37]. 

With respect to the factors described above, the registration method and position have the most significant influence on navigation accuracy. The investigation of the registration method on dynamic implant navigation is rare, which could directly impact the intraoperative navigation time and difficulty. Different DNS strategies utilize different registration methods. Paired-point coordinate transformation is the most widely used registration method [8,38,39]. It includes determining the coordinates of corresponding points (reference points) on the image (manually or semi-automatically on the computer) and the patient (using the probe of the navigation system) and calculating the geometric transformation of the best alignment of these points [40]. While occlusal splint registration is accurate and commonly used in DNS contexts, the fitting of the splint is critical and could result in deviations when conducted improperly. Some clinical researchers have pointed out that a high degree of accuracy can only reliably be achieved using bone-fixed fiducials, as dental- or mucosal-supported splints can give rise to deviations [40,41]. However, screw fixation inevitably exposes patients to additional trauma. Former research demonstrates the accuracy of DNS performed with occlusal splint registration [5,18,19,20]. For example, Chen et al. [33]. reported in vitro entry deviation, apex deviation, and angular deviation values of 1.07 ± 0.48 mm, 1.35 ± 0.55 mm, and 4.45 ± 1.97°, respectively, when using this approach. Cusp registration is an anatomical mark-based registration technique that functions similarly to bone marker registration when teeth are stable. In our prior study, we achieved similar levels of accuracy for both the cusp registration and U-tube registration approaches in the anterior maxillary and mandibular areas in vivo [42]. Under identical experimental conditions in the present study, we detected no significant differences in the relative accuracy of the U-tube and cusp registration techniques in the posterior area. The accuracy of cusp registration was also not influenced by the location of the missing teeth. When assessing the group with distal-extension absence, the stability of U-tube registration was found to be affected by the retention of the teeth and the mucosa, although this effect is likely to be more pronounced in vivo compared to in vitro. Cusp registration was a feasible approach that did not require the use of an additional registration device and was not impacted by the edentulous area. However, more studies of the cusp registration technique are warranted. As reported previously, it might also be affected by the operators’ experience [32,36,42]. Furthermore, if there were many metal restorations in the same jaw of missing teeth, the cusp registration was not a good choice [42]. Therefore, the advantages and limitations of this registration technique, as well as approaches to improving its accuracy, require further study.

In general, DNS accuracy studies are divided into model-based studies and clinical trials. Model-based research is an ideal method to evaluate the differences between systems as it eliminates many confounding factors associated with patient treatment, such as changes in bone mineral density, patient movement, and some variables related to imaging limitations. The presence of immature bone also hinders the display of bone and may affect the prediction of depth. In clinical trials, anatomical variations lead to large deviations from the plan. For these reasons, model-based research allows the direct comparison of the navigation accuracy of the system itself [32,36,43].

While these results are informative, there are several limitations of this study. First, this was an in vitro experiment, which was different from the clinical situation to some extent. However, efforts were made to mimic the clinical situation as closely as possible and many clinical confounding factors were controlled. Importantly, as the operator and the variables associated with anatomy (model, preclinical patient simulation, light conditions), surgery (drilling device, implant system, implant length, and diameter), and planning (CBCT, the software used for preoperative planning) were the same for all experiments, this study is better suited to directly evaluating the effects of the registration method on the accuracy of implant placement. In future studies, we will further explore the indications and specific approach parameters associated with cusp registration, particularly in the context of clinical application.

## 5. Conclusions

Dynamic computer-assisted surgery systems offer an invaluable approach to improve dental implant placement accuracy, and the results of this study indicate that both the U-tube and cusp registration methods can achieve accuracy in vitro. The cusp registration technique is a feasible approach and can also overcome several of the limitations of the U-tube registration technique without the need for an additional registration device. This approach is also more convenient for clinical use, highlighting its promising prospects as a method worthy of further clinical research.

## Figures and Tables

**Table 1:** Descriptive and bivariate results of the main outcome variables for both groups.

	All Enrolled	Cusp	U-Tube	*p* Value
N	64	32	32	
Angular deviation (°)	1.970 ± 1.042	2.118 ± 0.940	1.823 ± 0.999	0.141
Entry deviation (mm)	1.089 ± 0.515	1.172 ± 0.469	1.006 ± 0.567	0.320
Apex deviation (mm)	1.174 ± 0.531	1.23 ± 0.520	1.119 ± 0.561	0.126
EH (mm)	0.582 ± 0.358	0.554 ± 0.340	0.609 ± 0.378	0.544
AH (mm)	0.824 ± 0.429	0.738 ± 0.406	0.909 ± 0.442	0.112
ED (mm)	0.731 ± 0.529	0.780 ± 0.493	0.683 ± 0.583	0.476
AD (mm)	0.750 ± 0.574	0.791 ± 0.491	0.707 ± 0.660	0.567

EH: Entry Horizontal. AH: Apex Horizontal. ED: Entry Depth. AD: Apex Depth. No significant differences were observed (*p* > 0.05).

**Table 2:** Directions of horizontal deviation of the implant position obtained by the DNS-based method.

	Cusp		U-tube	
	Without Distal-Extension Absence (*n* = 16)	Distal-Extension Absence (*n* = 16)	Without Distal-Extension Absence (*n* = 16)	Distal-Extension Absence (*n* = 16)
Angular deviation (°)	1.957 ± 0.941	2.278 ± 0.941	1.675 ± 1.066	1.971 ± 1.265
*p* value	0.724		0.515	
Entry deviation (mm)	1.186 ± 0.512	1.158 ± 0.469	0.787 ± 0.459	1.224 ± 0.592
*p* value	0.323		0.026 *	
Apex deviation (mm)	1.260 ± 0.510	1.120 ± 0.544	0.868 ± 0.443	1.369 ± 0.567
*p* value	1		0.012 *	
EH (mm)	0.513 ± 0.242	0.595 ± 0.422	0.544 ± 0.269	0.685 ± 0.458
*p* value	0.505		0.263	
AH (mm)	0.663 ± 0.330	0.813 ± 0.468	0.895 ± 0.306	1.023 ± 0.530
*p* value	0.301		0.145	
ED (mm)	0.819 ± 0.574	0.741 ± 0.412	0.491 ± 0.400	0.875 ± 0.581
*p* value	0.663		0.061	
AD (mm)	0.832 ± 0.568	0.751 ± 0.416	0.494 ± 0.401	0.921 ± 0.802
*p* value	0.648		0.067	

* Statistically significant difference (*p* < 0.05).

## Data Availability

The data presented in this study are available on request from the corresponding author. The data are not publicly available due to privacy.
